# Fabrication of HfO_2 _patterns by laser interference nanolithography and selective dry etching for III-V CMOS application

**DOI:** 10.1186/1556-276X-6-400

**Published:** 2011-05-31

**Authors:** Marcos Benedicto, Beatriz Galiana, Jon M Molina-Aldareguia, Scott Monaghan, Paul K Hurley, Karim Cherkaoui, Luis Vazquez, Paloma Tejedor

**Affiliations:** 1 Instituto de Ciencia de Materiales de Madrid, CSIC. C/Sor Juana Inés de la Cruz 3, 28049 Madrid, Spain; 2 Instituto Madrileño de Estudios Avanzados de Materiales (Instituto IMDEA-Materiales). C/Profesor Aranguren, s/n. 28040 Madrid, Spain; 3 Tyndall National Institute, University College Cork, Lee Maltings Complex, Prospect Row, Cork, Ireland

## Abstract

Nanostructuring of ultrathin HfO_2 _films deposited on GaAs (001) substrates by high-resolution Lloyd's mirror laser interference nanolithography is described. Pattern transfer to the HfO_2 _film was carried out by reactive ion beam etching using CF_4 _and O_2 _plasmas. A combination of atomic force microscopy, high-resolution scanning electron microscopy, high-resolution transmission electron microscopy, and energy-dispersive X-ray spectroscopy microanalysis was used to characterise the various etching steps of the process and the resulting HfO_2_/GaAs pattern morphology, structure, and chemical composition. We show that the patterning process can be applied to fabricate uniform arrays of HfO_2 _mesa stripes with tapered sidewalls and linewidths of 100 nm. The exposed GaAs trenches were found to be residue-free and atomically smooth with a root-mean-square line roughness of 0.18 nm after plasma etching.

PACS: Dielectric oxides 77.84.Bw, Nanoscale pattern formation 81.16.Rf, Plasma etching 52.77.Bn, Fabrication of III-V semiconductors 81.05.Ea

## Introduction

Three-dimensional multi-gate field effect transistors with integrated mobility-enhanced channel materials (i.e. GaAs, In_x_Ga_1-x_As) and high-κ gate dielectrics (i.e. HfO_2_, Al_2_O_3_) are considered as plausible candidates to sustain Si complementary metal-oxide-semiconductor (CMOS) performance gains to and beyond the 22 nm technology generation in the next 5 to 7 years [1,2]. The rapid introduction of these new materials in non-planar transistor architectures will consequently have a high impact on front-end cleaning and etching processes. Cleaning processes thus need to become completely benign, in terms of substrate material removal and surface roughening. Moreover, high-κ gate etching offering high across-wafer uniformity, selectivity, and anisotropy will be essential to achieve a tight control over gate-length critical dimensions (CD) while keeping linewidth roughness low in future devices. To attain this goal, an adequate choice of photoresist type, etch bias power, and etch chemistry is necessary [3].

Patterning of HfO_2 _layers on Si substrates by means of different lithographic techniques and dry etching in F-, Cl-, Br-, CH_4_-, and CHF_3_-based plasma chemistries has been extensively investigated [4-7]. Comparatively much less attention has been paid to patterning ultrathin layers of HfO_2 _deposited on GaAs substrates despite its key role in the fabrication of next generation non-planar high-κ/III-V transistors. In recent papers, we have studied the nanoscale patterning of HfO_2_/GaAs by electron beam lithography and inductively coupled plasma reactive ion etching (ICP-RIE) using BCl_3_/O_2 _and SF_6_/Ar chemistries [8,9]. Only the less-reactive F-based chemistry showed good etch selectivity of HfO_2 _over GaAs (i.e. 1.5) and adequate control of the etching rate. In this letter, we report on the fabrication of nanopatterned HfO_2 _ultrathin layers on GaAs substrates by laser interference nanolithography (LInL) and selective ICP-RIE in a CF_4 _plasma chemistry. The main HfO_2 _etching characteristics studied by a combination of atomic force microscopy (AFM), high-resolution scanning electron microscopy (HR-SEM), and high-resolution transmission electron microscopy (HR-TEM)/energy-dispersive X-ray spectroscopy microanalysis (EDS) are presented, with specific emphasis on pattern resolution; etch profile; and GaAs surface roughness and composition.

## Experimental

All experiments described here were performed on 10-nm-thick HfO_2 _layers grown by atomic layer deposition (Cambridge NanoTech Inc., Cambridge, MA, USA) on a 2-in.-diameter GaAs (001) wafer (Wafer Technology Ltd., Milton Keynes, UK), where a 400-nm-thick GaAs buffer layer had been previously deposited by metal-organic vapour phase epitaxy. Nanostructuring of the HfO_2 _thin film was carried out by Lloyd's mirror LInL using a commercial system (Cambridge NanoTools LLC, Somerville, MA, USA) and a He-Cd laser (λ = 325 nm) as the light source. Prior to exposure to the laser source, the HfO_2_/GaAs substrates were first spin coated with a 210-nm-thick antireflective coating (ARC), then covered by a 20-nm-thick SiO_2 _layer grown by plasma-enhanced chemical vapour deposition, and finally spin coated with a negative photoresist (OHKA PS4, Tokyo OHKA Kogyo Co., Japan). The ARC has the adequate refractive index to suppress 325-nm reflections from the substrate. The SiO_2 _layer acts as a mask and improves the pattern transfer from the photoresist to the ARC. Subsequently, a stripe pattern was transferred to the photoresist by LInL. The samples were then introduced in an ICP reactive ion etcher (PlasmaLab80Plus-Oxford Instruments, Oxfordshire, UK) to transfer the pattern to the HfO_2 _layer through a series of successive etching steps aimed to selectively remove the exposed areas of SiO_2_, ARC, and HfO_2_. An initial CF_4 _plasma-etching step was used to transfer the pattern from the resist to the SiO_2 _layer. This was followed by O_2 _plasma etching to transfer the pattern from the SiO_2 _to the ARC. During this step, the resist layer is completely eliminated. Finally, the HfO_2 _was patterned in a CF_4 _plasma using a radio-frequency power of 100 W. The nanostructured HfO_2_/GaAs samples were then exposed to a second treatment with O_2 _plasma to eliminate all organic residues from the surface. Finally, a dip in a 1:1 HCl/H_2_O solution followed by a D.I. H_2_O rinse was applied to clean the exposed GaAs bottom trenches.

The surface morphology of the patterned HfO_2_/GaAs samples was examined with an AFM microscope (5500 Agilent, Santa Clara, CA, USA) working in the dynamic mode. Si cantilevers (Veeco, Plainview, NY, USA) with a nominal radius of 10 nm were used. An SEM microscope (FEI NovaNanoSEM 230, FEI Co., Hilsboro, OR, USA) was used for HR-SEM sample examination. Cross-sectional specimens suitable for HR-TEM were prepared using a focused ion beam (FIB) FEI Quanta FEG dual-beam system (FEI Co.). In order to protect the surface of interest from milling by the Ga^+ ^ion beam during sample preparation, a Pt layer was deposited in the FIB on the HfO_2_/GaAs nanopatterns. This common procedure is accomplished by introducing an organometallic gas in the vacuum chamber, where it decomposes on the sample surface upon interaction with the ion beam. HR-TEM/EDS compositional maps were acquired using a Philips Tecnai 20 FEG TEM (FEI Co.) operating at 200 keV.

## Results and discussion

The main characteristics of the nanostructuring process were investigated by a combination of AFM, HR-SEM, HR-TEM, and EDS. In particular, we studied the resolution and anisotropy of the HfO_2_-etched nanostructures as well as the roughness and compositional integrity of the underlying GaAs surface.

**Figure 1:**
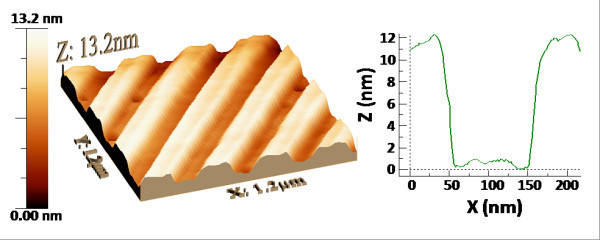
**AFM images of the HfO_2 _nanopattern**. (**a**) Three-dimensional view of the nanostructured HfO_2_/GaAs surface morphology. (**b**) Cross-section scan profile of an etched trench.

The surface morphology of the as-deposited and nanostructured HfO_2_/GaAs samples was examined by AFM. The root-mean-square (r.m.s.) surface roughness (σ) extracted from 2 × 2-μm AFM images was found to be 0.7 ± 0.01 nm for the as-deposited HfO_2 _film and 4.9 ± 0.01 for the nanostructured HfO_2_/GaAs sample. Figure 1 depicts a three-dimensional image (1.2 × 1.2 μm) of the HfO_2_/GaAs surface topography after nanostructuring and a typical scan profile across an etched trench. The latter revealed the formation of a tapered sidewall due to directional chemical etching and the presence of re-deposited reaction by-products on the edges of the HfO_2 _mesa stripes. The values of the r.m.s. line roughness (*R*_a_) measured along the HfO_2 _stripes and the etched GaAs trenches were 0.14 ± 0.03 nm and 0.18 ± 0.03 nm, respectively. The value of the GaAs line roughness measured in this work is comparable to that reported previously for HfO_2 _etching using a SF_6_/Ar plasma (0.13 nm) [8]. Etching with a CF_4 _plasma chemistry thus provides an atomically smooth GaAs surface, which is a critical requirement for subsequent selective III-V growth during device fabrication. In fact, preliminary III-V molecular beam epitaxy experiments to be reported elsewhere indicate that both the quality of the starting GaAs surface and the inclined sidewalls of the HfO_2 _nanopatterns are adequate for selective area growth and the resulting III-V nanostructures do not suffer from microtrench formation near the high-κ gate oxide, reported by other authors [10].

**Figure 2:**
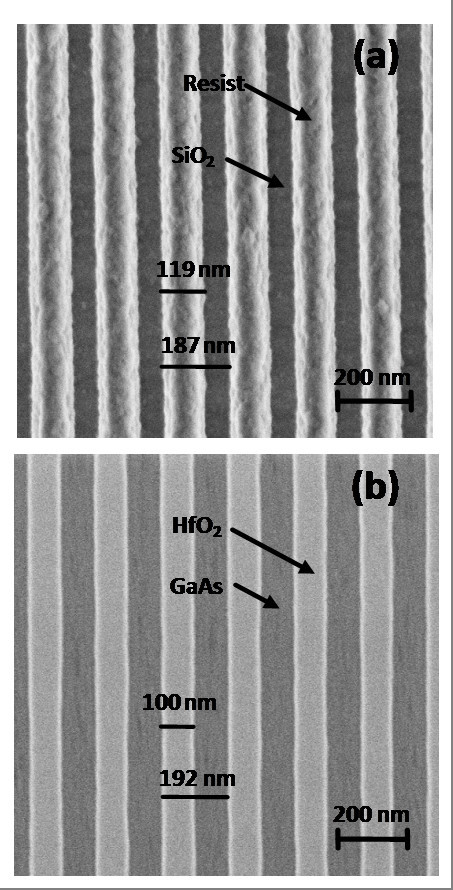
**HR-SEM images of the resist and HfO_2 _patterns**. Plan view images of (**a**) the resist pattern after laser interference nanolithography and (**b**) the resulting HfO_2 _nanopattern after CF_4_/O_2 _ICP-RIE and HCl/H_2_O cleaning.

Pattern transfer to the HfO_2 _ultra thin film was investigated by HR-SEM. The 1.3 × 1.3-μm scanning electron micrographs in Figure 2 illustrate the sample morphology at two different stages of the patterning process. Figure 2a is a plan view of the sample surface after laser lithography showing the patterned resist stripes and the underlying SiO_2 _layer. The average values of the resist linewidth and the pitch are 119 ± 6 nm and 187 ± 6 nm, respectively. The micrograph depicted in Figure 2b is a plan view of the nanostructured surface after exposure to the sequence of CF_4 _and O_2 _plasma steps and the final HCl/H_2_O surface cleaning described above. The image shows well-defined HfO_2_-etched features on the GaAs substrate. Moreover, no evidence of HfO_2 _residues on the groove bottom was found when a backscattered electron detector was used to enhance the compositional contrast in the image. The average HfO_2 _linewidth and pitch of the nanopattern, measured from Figure 2b, were 100 ± 7 nm and 192 ± 6 nm, respectively.

**Figure 3:**
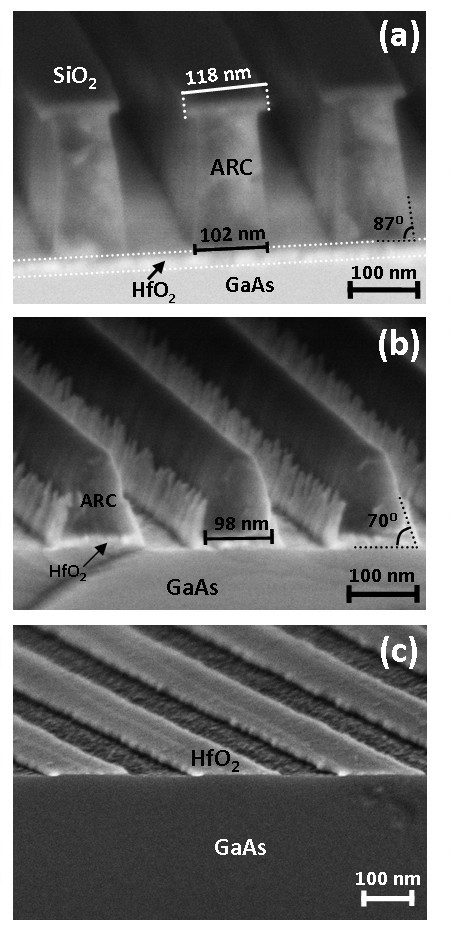
**HR-SEM images of the pattern transfer process**. (**a**) Cross-section view of the etched multilayer structure after pattern transfer to the SiO_2 _and ARC layers. (**b**) Cross-section view of the structure after pattern transfer to the HfO_2 _layer, showing re-deposition of reaction by-products on the sidewalls. (**c**) View of the nanostructured HfO_2 _stripes.

In order to elucidate the origin of the linewidth narrowing observed in the HfO_2 _stripes with respect to the original resist pattern, a more detailed study of the intermediate etching steps was undertaken. These were characterised by analysing cross-sectional HR-SEM images of the sample at different stages of the nanostructuring process. Figure 3a depicts the cross-section of the sample after pattern transfer to the SiO_2 _and ARC layers, showing that the SiO_2 _linewidth (118 nm) has not varied significantly with respect to that of the resist pattern. In addition, the etched sidewalls are vertical, hence, indicating that the pattern was precisely transferred to the SiO_2 _layer during the first CF_4 _etching step. By contrast, O_2 _plasma etching of the ARC layer proceeds with undercut and inclined sidewall (87°) formation, suggesting that some interaction between radicals from the gas phase and the sidewalls has occurred. The linewidth at the bottom of the ARC is consequently reduced (102 nm) with respect to the original resist pattern, as shown in the image.

**Figure 4:**
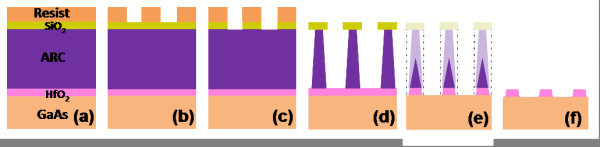
**Schematic of the HfO_2 _nanostructuring process**. (**a**) Schematic drawing of the starting multilayer structure. (**b**) Patterning of the photoresist by laser interference lithography. (**c**) Pattern transfer to the SiO_2 _layer by CF_4 _ICP-RIE. (**d**) Pattern transfer to the ARC by O_2 _ICP-RIE. (**e**) Selective ICP-RIE of the HfO_2 _layer with CF_4_. (**f**) Elimination of the ARC with O_2 _ICP-RIE and final cleaning with HCl/H_2_O.

Figure 3b illustrates the sample cross-section after HfO_2 _selective etching with CF_4_. This process has been estimated to occur at a rate of 0.06 nm/s. Such slow HfO_2 _etching rate is advantageous with respect to previous reports using SF_6_/Ar [8] from the process control viewpoint, as it allows to process a typical 2-nm-thick gate oxide in a practicable etching time, i.e. approximately 30 s. As shown in the image, a tapered etch profile with a 70° inclination angle is achieved by the formation of a sidewall passivation layer comprised of non-volatile reaction by-products of the CF_4 _etching process. It should be noted here that the patterned resist mask had been completely eliminated during the previous O_2 _plasma treatment and, consequently, the exposed SiO_2 _stripes and the ARC layer are gradually etched by the CF_4 _plasma during pattern transfer to the HfO_2 _film. This contributes to a further reduction of the pattern linewidth and to the formation of an HfO_2 _foot on both mesa edges, which is only observable by HR-TEM (see below). The width of the HfO_2 _mesa top measured from Figure 3b was 98 nm at this stage of the process. The width of the mesa bottom could not be determined from the same image due to the presence of re-deposited material. Notwithstanding, we have estimated that the bottom linewidth is approximately 105 nm, taking into account that the 70° ARC sidewall inclination is transferred to the HfO_2 _layer without any significant variation. Comparison of this value with the final dimension of the HfO_2 _stripes (Figure 3c), i.e. 100 nm, suggests that the last HCl/H_2_O wet etch further contributes to narrow the linewidth. The schematic diagram shown in Figure 4 illustrates the HfO_2 _nanofabrication process flow.

**Figure 5:**
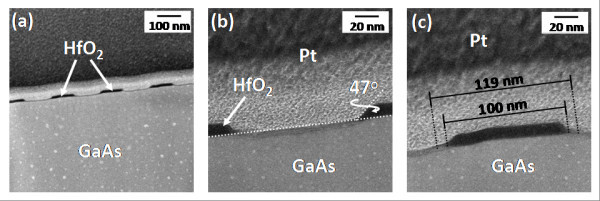
**HR-TEM images of the pattern transfer process**. (a) Bright-field cross-section image of the periodic HfO_2 _stripe pattern. (b) Close-up view of an etched trench. The GaAs surface structure appears modified by the plasma etch. The formation of a sloped sidewall can also be seen. (c) Close-up view of a 100-nm-wide HfO_2 _mesa stripe. The formation of an approximately 10-nm-wide foot due to mask erosion is observed on both sides of the HfO_2 _mesa.

**Figure 6:**
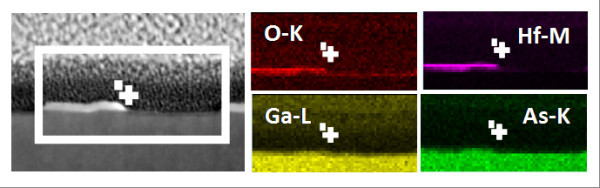
**TEM-EDS analysis of the HfO_2_/GaAs pattern**. (**a**) Cross-section TEM image of a 100nm-wide HfO_2 _mesa stripe and a GaAs trench after nanostructuring. (**b**) Corresponding EDS elemental maps for O (K), Hf (M), Ga (L), and As (L). The amorphous layer located at the trench bottom surface is constructed of gallium oxide. Hf is concentrated in the mesa stripe and side feet.

The structure of the nanopatterned HfO_2_/GaAs samples was investigated by HR-TEM. Figure 5a, b, c depicts a series of cross-section HR-TEM images showing the periodic HfO_2 _nanopattern fabricated on the GaAs epilayer as well as details of an etched trench and a typical HfO_2 _mesa stripe. The anisotropic nature of the etch profile and the existence of slight variations in sidewall inclination are observable in these images. The HfO_2 _sidewall angle measured from Figure 5b, i.e. 47°, contrasts with that measured after CF_4 _etching, i.e. 70°. The HCl/H_2_O wet etch step thus appears to alter both the HfO_2 _linewidth and the mesa profile. In addition, Figure 5c clearly shows the formation of a approximately 10-nm-long foot at either side of the HfO_2 _stripe, due to the progressive erosion of the ARC and SiO_2 _layers during CF_4 _etching mentioned above. Note that the total HfO_2 _width, including the feet at both sides of the mesa, corresponds roughly to the resist linewidth in the original pattern, as indicated in the figure. The HfO_2_/GaAs interface appears quite abrupt and the underlying GaAs substrate shows no evidence of lattice damage. Nevertheless, an approximately 5-nm-thick amorphous layer is observed in the exposed GaAs regions (Figure 5b), which is likely to have formed as a result of ion damage or oxidation during exposure to the CF_4 _and O_2 _plasmas. Further investigation of the chemical composition of the HfO_2_/GaAs samples was performed by TEM/EDS analysis. The cross-sectional elemental maps corresponding to O (K), Hf (M), Ga (L), and As (K), gathered in Figure 6, indicate that the sub-surface layer is mainly constructed of gallium oxide, the less volatile of the oxidation products of GaAs, which is formed during the final exposure to the O_2 _plasma. This oxide layer can be removed prior to epitaxy by standard thermal desorption at 600°C. Finally, the composition map corresponding to Hf (M) shows that Hf is concentrated in the mesa stripes, although traces of this element were also detected in the mesa foot.

## Conclusions

We have demonstrated the fabrication of HfO_2_/GaAs patterns with nanoscale resolution using He-Cd laser interference lithography and dry etching using a combination of CF_4 _and O_2 _plasmas. The etched GaAs trenches formed by this process were found to be residue-free and atomically smooth after plasma etching. Strong sidewall passivation during HfO_2 _selective etching and wet cleaning with an HCl/H_2_O solution results in the formation of tapered HfO_2 _etch profiles. Optimisation of the CF_4 _plasma composition and etch bias power to lessen the re-deposition of non-volatile by-products, in combination with the use of more benign cleaning solutions than HCl/H_2_O, are some of the future improvements to be introduced in the current process to reach the approximately 30 nm HfO_2 _gate lengths and CD control better than 2 nm required for the fabrication of III-V-based CMOS.

## Competing interests

The authors declare that they have no competing interests.

## Authors' contributions

MB performed the statistical analysis, participated in the interpretation of data, and drafted the manuscript. BG carried out the TEM characterization and participated in the interpretation of the data. JMMA carried out the TEM sample preparation and analysis. SM, PKH, and KC participated in the deposition of the GaAs and HfO_2 _layers. LV was responsible for AFM characterization. PT conceived the study, participated in the interpretation of data, and wrote the manuscript. All authors read and approved the final manuscript.

## References

[B1] Skotnicki T Fenouillet-Beranger C Gallon C Bœuf F Monfray S Payet F Pouydebasque A Szczap M Farcy A Arnaud F Clerc S Sellier M Cathignol A Schoellkopf JP Perea E Ferrant R Mingam H Innovative materials, devices, and CMOS technologies for low-power mobile multimedia IEEE Trans Electron Devices 2008 55 96 130

[B2] Radosavljevic M Dewey G Fastenau JM Kavalieros J Kotlyar R Chu-Kung B Liu WK Lubyshev D Metz M Millard K Mukherjee N Pan L Pillarisetty R Rachmady W Shah U Chau R Non-planar, multi-gate InGaAs quantum well field effect transistors with high-k gate dielectric and ultra-scaled gate-to-drain/gate-to-source separation for low power logic applications Proceedings of the IEEE International Electron Devices Meeting (IEDM) 6-8 December 2010; San Francisco 2010 IEEE 6.1.1 6.1.4

[B3] The International Technology Roadmap for Semiconductors http://www.itrs.net 2009 edition

[B4] Norasetthekul S Park PY Baik KH Lee KP Shin JH Jeong BS Shishodia V Norton DP Pearton SJ Etch characteristics of HfO_2 _films on Si substrates Appl Surf Sci 2002 187 75 81 10.1016/S0169-4332(01)00792-9

[B5] Kitagawa T Nakamura K Osari K Takahashi K Ono K Oosawa M Hasaka S Inoue M Etching of High-*k *Dielectric HfO_2 _Films in BCl_3_-Containing Plasmas Enhanced with O_2 _Addition Jpn J Appl Phys 2006 45 L297 L300 10.1143/JJAP.45.L297

[B6] Sungauer E Mellhaoui X Pargon E Joubert O Plasma etching of HfO_2 _in metal gate CMOS devices Microelectron Eng 2009 86 965 967 10.1016/j.mee.2008.10.026

[B7] Park JB Lim WS Park BJ Park IH Kim YW Yeom GY Atomic layer etching of ultra-thin HfO_2 _film for gate oxide in MOSFET devices J Phys D: Appl Phys 2009 42 055202 055207 10.1088/0022-3727/42/5/055202

[B8] Anguita J Benedicto M Álvaro R Galiana B Tejedor P Nanoscale Selective Plasma Etching of Ultrathin HfO_2 _Layers on GaAs for Advanced Complementary Metal-Oxide-Semiconductor Devices Jpn J Appl Phys 2010 49 106504 106507 10.1143/JJAP.49.106504

[B9] Benedicto M Anguita J Alvaro R Galiana B Molina-Aldereguia JM Tejedor P Nanostructuring of ultra-thin HfO_2 _layers for high-κ/III-V device application J Nanosci Nanotechnol 2011 11 1 5 10.1166/jnn.2011.3498 22400270

[B10] Burek GJ Wistey MA Singisetti U Nelson A Thibeault BJ Bank SR Rodwell MJW Gossard AC Heigth-selective etching for regrowth of self-aligned contacts using MBE J Cryst Growth 2009 311 1984 1987 10.1016/j.jcrysgro.2008.11.012

